# Vocal cord collapse during phrenic nerve-paced respiration in congenital central hypoventilation syndrome

**DOI:** 10.12688/f1000research.1-42.v1

**Published:** 2012-11-01

**Authors:** Mark C Domanski, Diego A Preciado

**Affiliations:** 1 Department of Surgery, University of Mississippi, Jackson, MS, 39216, USA; 2 Department of Otolaryngology, Children’s National Medical Center, Washington, D.C., 20010-2970, USA

## Abstract

**Objective: **Phrenic nerve pacing can be used to treat congenital central hypoventilation syndrome (CCHS). We report how the lack of normal vocal cord tone during phrenic paced respiration can result in passive vocal cord collapse and produce obstructive symptoms.

**Methods: **We describe a case of passive vocal cord collapse during phrenic nerve paced respiration in a patient with CCHS. As far as we know, this is the first report of this etiology of airway obstruction. The patient, a 7-year-old with CCHS and normal waking vocal cord movement, continued to require nightly continuous positive airway pressure (CPAP) despite successful utilization of phrenic nerve pacers. On direct laryngoscopy, the patient’s larynx was observed while the diaphragmatic pacers were sequentially engaged.

**Results:** No abnormal vocal cord stimulation was witnessed during engaging of either phrenic nerve stimulator. However, the lack of normal inspiratory vocal cord abduction during phrenic nerve-paced respiration resulted in vocal cord collapse and partial obstruction due to passive adduction of the vocal cords through the Bernoulli effect. Bilateral phrenic nerve stimulation resulted in more vocal cord collapse than unilateral stimulation.

**Conclusions:** The lack of vocal cord abduction on inspiration presents a limit to phrenic nerve pacers.

## Introduction

Congenital central hypoventilation syndrome (CCHS) is a rare disorder typified by the lack of ventilatory responsiveness to hypoxemia and hypercarbia. The patient with CCHS will typically have adequate conscious control of breathing when awake. However, when asleep, the anatomic nervous system fails to maintain respiration. Patients with CCHS may have other disorders of the autonomic nervous system such as Hirschsprung’s disease, lack of heart rate variability, poor temperature regulation, and diminished pupillary light response. Tumors of neural crest origin such as ganglioneuromas, neuroblastomas and ganglioneuroblastomas are also associated with CCHS ^1^.

CCHS is a rare clinical entity. CCHS was first described in 1970s by Mellins *et al.* ^2^. As of 1999, it was estimated that there were only 200–300 patients with CCHS worldwide ^3^.

CCHS is caused by a heterogeneous mutation in PHOX2B. PHOX2B is highly conserved transcription factor found on chromosome 4p12. PHOX2B is expressed in both the central and peripheral autonomic nervous system during human development. A mouse model of CCHS showed that Phox2B -/- mice fail to develop the normal neuronal connections of several structures including the solitary tract ^4, 5^.

Children with CCHS typically present soon after birth with duskiness or cyanosis upon falling asleep. During sleep, falling oxyhemoglobin saturation and rising carbon dioxide levels fail to increase respiration or awaken the infant. The differential diagnosis includes discrete congenital myopathy, myasthenia gravis, altered airway anatomy, diaphragm dysfunction, congenital cardiac disease, a structural hindbrain or brainstem abnormality, Mobius syndrome, and inborn errors of metabolism ^1^.

Evaluation of suspected CCHS may include a muscle biopsy, chest x-ray, fluoroscopy of the diaphragm, bronchoscopy, electrocardiogram, Holter recording, echocardiogram, magnetic resonance imaging of the brain and brainstem, serum and urinary carnitine levels. Ophthalmological evaluation should assess for pupillary reactivity and optic disk anatomy. A rectal biopsy should be considered because of the association with Hirschsprung’s disease ^1^.

Treatment of the respiratory compromise in CCHS consists of a tracheostomy and adequate ventilatory support. Because of the persistent lack of response to hypoxemia and hypercarbia, frequent monitoring of pulse oximetry and end tidal CO _2_ may be prudent ^1^. Once the child reaches appropriate age, insertion of diaphragmatic pacers may be considered ^6^. Diaphragmatic pacers work by stimulating the phrenic nerve in the chest, resulting in diaphragm contraction and respiration. The energy for stimulation is provided by an external portable power source. Successful use of diaphragmatic stimulation may in some cases allow for tracheal decannulation ^1^.

Normal respiration is more than just appropriate diaphragmatic response to hypoxemia and hypercarbia. During inspiration, the vocal cords abduct. Classically, this "V-shaped" aperture is called the glottic "chink" ^7^. If the vocal cords were not actively abducted during inspiration, they would be drawn together via the Bernoulli effect ^8^.

## Case Description

A seven year-old female presented with a history of CCHS and a current complaint of obstructive sleep apnea requiring continuous positive airway pressure (CPAP).

In the newborn period, the patient had been managed with a tracheostomy and traditional ventilatory support. An extensive workup failed to demonstrate any other major developmental abnormalities. In the preschool years, she received bilateral placement of intrathoracic diaphragmatic pacers that eventually allowed decannulation.

During the day the patient ambulated with an external power source that was programmed to stimulate the pacing wires if she did not take any breaths in a predefined time period. This was in case the patient accidentally fell asleep. At night the device was set to provide her with continuous respirations. However, at night, the patient also required continuous positive airway pressure because of an obstructive component of her sleep apnea.

**Figure 1.**
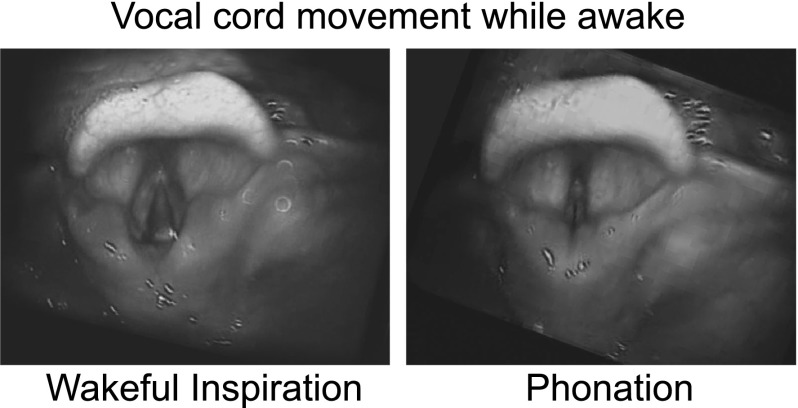
Flexible laryngoscopy showed normal function while awake.

Flexible nasal laryngoscopy showed no adenotonsillar hypertrophy. Pharyngeal and tongue anatomy was normal. Vocal cords movement was normal, including abduction during inspiration (Figure 1).

**Figure 2.**
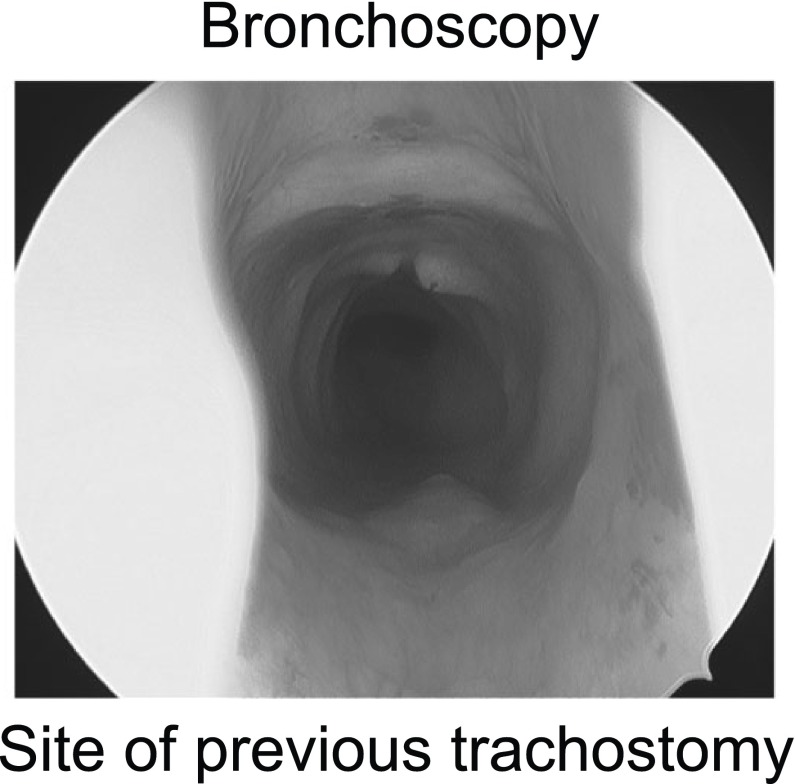
Bronchosopy failed to identify any site of lower airway obstruction.

The patient was scheduled for evaluation in the operating room under general anesthesia. The patient received a preoperative dose of oral midazolam. In the operating room the phrenic pacers were turned off and general anesthesia was induced using inhalational agents. Muscle relaxants were not used. The patient was masked without difficulty. Direct laryngoscopy was performed without an endotracheal tube in place. No laryngeal structural pathology was found. Bronchoscopy using a rigid 4 mm Hopkins rod showed a well-healed tracheostomy site without any granulation or tracheomalacia (Figure 2).

**Figure 3.**
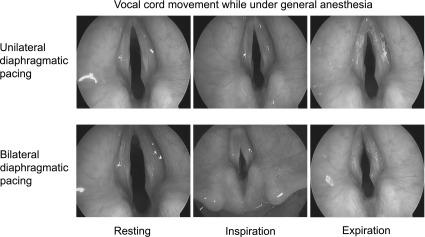
Direct laryngoscopy while asleep and breathing via diaphragmatic pacing. Vocal cord collapse was greater during bilateral than unilateral diaphragmatic pacing.

Because of the patient’s diaphragmatic pacers, it was possible to observe how the patient "normally" breathed while asleep. While performing direct laryngoscopy, we proceeded to turn on the right and left phrenic nerve pacers individually and then together. Unilateral stimulation of the diaphragm resulted in respiration. No active stimulation such as myoclonus, abduction or abduction of the vocal cords was noted. However, during inspiration the true vocal cords were pulled medially. This was most evident at the start of inspiration. Expiration resulted in flutter of the vocal cords most prominent at the anterior commissure. Bilateral phrenic nerve stimulation produced greater respiratory efforts. This was accompanied by greater medial pull of the true vocal cords during inspiration along with audible stridor. Flutter of the vocal cords on expiration was greater as well (Figure 3).

Once the examination was complete, the patient was awoken in the operating room. The patient’s phrenic nerve pacers were turned on while positive pressure was provided via masking – allowing the child to breath while still under anesthesia. As the child awoke, she gradually took voluntary control of her respiration at which time the phrenic pacers automatically ceased.

## Conclusion

CCHS is a rare, multifaceted disorder involving severe central sleep apnea. Treatment is supportive, including aggressive respiratory support such a tracheostomy and sleep-time ventilatory support. Phrenic nerve pacing can obviate the need for external ventilator support, allowing the patient to be considered for decannulation. However, decannulation means that during sleep, air will pass by the vocal cords. As demonstrated in this case report, the vocal cords do not function normally in phrenic nerve-paced respiration. Because there is no central respiratory drive, there is no stimulation for the vocal cords to abduct on inspiration. Instead, during phrenic nerve pacer respiration, the vocal cords are drawn together via the Bernoulli effect and obstructive sleep apnea can result.

As many children with CCHS develop sequelae compatible of intermittent hypoxemia, passive True Vocal Chord (TVC) collapse is important to consider when evaluating their sleep apnea. The lack of normal vocal cord abduction on inspiration presents a limitation to diaphragmatic paced respiration. Our patient required CPAP while asleep for this reason.
