# Supplementary material for: Vocal cord collapse during phrenic nerve-paced respiration in congenital central hypoventilation syndrome
Source: F1000Res. 2012 Nov 1;1:42. [Version 1] doi: 10.12688/f1000research.1-42.v1 (PMC3752620; doi:10.12688/f1000research.1-42.v1)
Supplement: Peer review history

# Peer review: Referee response for version 1

**Published:** 2012-11-11

**Authors:** Anastasios G Hantzakos

**Affiliations:** 1st Department of Orolaryngology, Head and Neck Surgery, University of Athens, Hippocrateion General Hospital, Athens, Greece

Interesting paper! It would be further appreciated if the authors could add a section labeled “Discussion” where the details on the principles of PNP respiration and proposed solutions to prevent obstruction could be noted.

I have read this submission. I believe that I have an appropriate level of expertise to confirm that it is of an acceptable scientific standard.

# Peer review: Referee response for version 1

**Published:** 2012-11-02

**Authors:** Nina Shapiro

**Affiliations:** Division of Head and Neck Surgery, University of California, Los Angeles School of Medicine, Los Angeles, CA, USA

This is an excellent review of a rare disorder, Congenital Central Hypoventilation Syndrome. The authors present a thorough presentation of the nature of this disorder, followed by an illustrative case of a patient who developed paradoxical vocal cord mobility during phrenic nerve stimulation.

Utilization of phrenic nerve-paced respiration is carried out in effort to obviate the need for tracheotomy tube placement in these patients. However, the authors noted that this technique can lead to absence of vocal cord abduction on inspiration, which in turn leads to vocal fold closure and airway obstruction, secondary to the Bernouilli effect. Intraoperative photos are provided, as well as the outcome for this, and potentially future patients seen with this disorder.

I have read this submission. I believe that I have an appropriate level of expertise to confirm that it is of an acceptable scientific standard.
